# Interactions of Cells and Biomaterials for Nerve Tissue Engineering: Polymers and Fabrication

**DOI:** 10.3390/polym15183685

**Published:** 2023-09-07

**Authors:** Meaghan E. Harley-Troxell, Richard Steiner, Rigoberto C. Advincula, David E. Anderson, Madhu Dhar

**Affiliations:** 1 Tissue Engineering and Regenerative Medicine, Large Animal Clinical Sciences, College of Veterinary Medicine, University of Tennessee, Knoxville, TN 37996, USA; mharley4@vols.utk.edu (M.E.H.-T.); rcsteiner925@gmail.com (R.S.); dander48@utk.edu (D.E.A.); 2 Department of Chemical and Biomolecular Engineering, University of Tennessee, Knoxville, TN 37996, USA; radvincu@utk.edu; 3 Oak Ridge National Laboratory, Center for Nanophase Materials Sciences, Oak Ridge, TN 37831, USA

**Keywords:** nerve tissue engineering, biocompatibility, polymers, biomaterials, nanomaterials, hydrogels, 3D printing

## Abstract

Neural injuries affect millions globally, significantly impacting their quality of life. The inability of these injuries to heal, limited ability to regenerate, and the lack of available treatments make regenerative medicine and tissue engineering a promising field of research for developing methods for nerve repair. This review evaluates the use of natural and synthetic polymers, and the fabrication methods applied that influence a cell’s behavior. Methods include cross-linking hydrogels, incorporation of nanoparticles, and 3D printing with and without live cells. The endogenous cells within the injured area and any exogenous cells seeded on the polymer construct play a vital role in regulating healthy neural activity. This review evaluates the body’s local and systemic reactions to the implanted materials. Although numerous variables are involved, many of these materials and methods have exhibited the potential to provide a biomaterial environment that promotes biocompatibility and the regeneration of a physical and functional nerve. Future studies may evaluate advanced methods for modifying material properties and characterizing the tissue–biomaterial interface for clinical applications.

## 1. Introduction

The nervous system is divided into two distinct components: the central nervous system (CNS), composed of the brain and the spinal cord, and the peripheral nervous system (PNS), composed of the cranial and spinal nerves that extend from the CNS throughout the body. The normal, healthy nervous system is responsible for sensory, motor, and cognitive functions [1]. Neural injuries and diseases disrupt the signaling pathways responsible for these everyday acts, ultimately declining an individual’s quality of life. Affecting millions of people in the United States (US) alone, neural injuries include both CNS injuries, such as traumatic brain injuries (TBIs), spinal cord injuries (SCIs), and neurodegenerative diseases (NDs), as well as peripheral nerve injuries and degeneration (PNI) [2,3,4,5,6]. Each injury has a classification system identifying the nerve damage level [7,8,9]. In the PNS, a lower degree of injury will typically regenerate independently. However, more severe injuries in the PNS and most injuries in the CNS cannot heal without assistance.

A severe PNI is one in which, in addition to the axon and myelin sheath, the tissues surrounding the various bundles of nerve cells (endoneurium, perineurium, and epineurium) are also damaged, and the size of the PNI gap is more significant than 10 mm. Once the endoneurium (the innermost tissue layer) is affected, the injury worsens when the elasticity causes the proximal and distal nerve ends to retract. Widening the gap makes it more difficult for the developing axon to align accurately and reinnervate the target tissue to restore nerve function. The degenerative process that fragments the cytoskeleton of the axon causes cellular debris to accumulate in the affected area alongside the damaged cells from the initial injury. Secreted pro-inflammatory cytokines, immune cells, and harmful reactive oxygen intermediates (ROIs) in the environment further impair the regenerative ability. The process ends when the nerve stumps develop dense fibrous scar tissue, rendering self-renewal no longer possible [10,11,12,13,14,15].

While macrophages and Schwann cells help clear debris and direct cell growth in the PNS, their lack of involvement in the brain and spinal cord damages their capacity to regenerate. Like severe PNIs, most CNS injuries result in a sustained inflammatory state filled with cellular debris and impaired by scar tissue. Astrocytes, a supportive glial cell, propagate neuroinflammation and aggregate to form a glial scar preventing self-renewal. The CNS also involves the blood–brain barrier (BBB), whose role is to prevent cells, proteins, and large molecules circulating in the bloodstream from entering the CNS to protect these tissues from harm and shield the sensitive ecosystem. While some CNS injuries damage the BBB with initial insult, others cause secondary injury from the prolonged presence of inflammatory factors that exacerbate cell death, which may occur minutes to months after the original trauma. This disruption allows the peripheral immune cells and exogenous proteins to intrude into the already hostile environment where they are generally absent, further destabilizing the area [11,13,14,15].

Ultimately, both of these processes have the same result, a microenvironment unsuitable for regeneration, perpetuating disruption of regular nervous system functions [16]. Available treatments include various surgical and non-surgical options, though all are limited in restoring full neural function. The gold standard for a PNI is the use of autografts. Clinical use of autografts fails to restore full function in up to 33% of patients and includes the risk associated with the harvest surgery, loss of innervation, scarring and neuroma formation at the donor site, and limited tissue availability [7,11,17]. CNS treatment options are even more limited, focusing on symptom management to limit the effects of the secondary injury [18,19].

**Figure 1:**
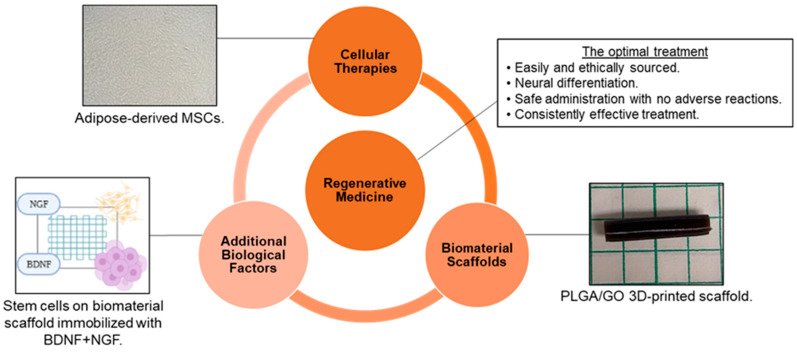
Tissue engineering and regenerative medicine can provide conditions that support physical and functional nerve regeneration. The influence of material and design on cell interactions must be considered to generate the optimal treatment [Images from Harley-Troxell, Dhar, unpublished data; figure made with BioRender and Microsoft PowerPoint].

As a result of the limited ability of peripheral nerves to regenerate without assistance and the lack of availability of efficient therapies for nerve repair, tissue engineering and regenerative medicine have become promising areas of study to develop novel, effective treatments. The goal of tissue engineering and regenerative medicine is to use exogenous cells, biomaterials, and/or additional biological factors to promote a more favorable microenvironment after an injury or disease so that the tissue can be repaired, and function restored. Although reaching this goal is complicated by the intricacies of the nervous system, optimal treatment for neural applications would be: (1) tissues obtained from a source that is easy and ethical to access; (2) therapeutic materials that can promote differentiation into specific neural and glial cell types; (3) a method of administration that is safe, minimally invasive, and causes no short-term or long-term adverse effects; and (4) an effective treatment that fully and consistently restores function [15,20,21] (Figure 1). To achieve this goal and optimize the scaffold design, consideration of the biomaterial–cell interactions and the constructs’ influence on cell behavior is required. This review will evaluate the natural and synthetic polymers that form the construct, as well as the fabrication methods that modify the material properties that influence cell behavior. This review will also evaluate the methods to measure cell response in vitro and *in vivo*, and the endogenous and exogenous cell types that will interact with the developed construct.

## 2. Biocompatibility Assessment

**Figure 2:**
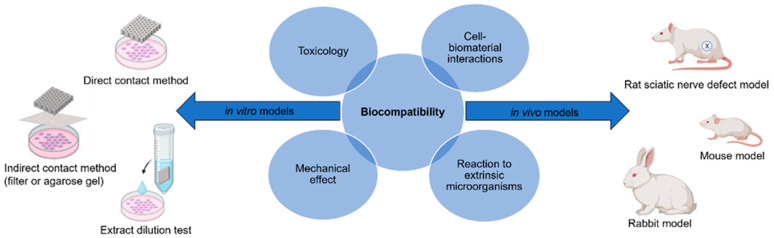
Biocompatibility can be evaluated using the appropriate in vitro and in vivo models to ensure there is no toxicology, mechanical effects, reaction to extrinsic microorganisms, or cell–biomaterial interactions [figure made with BioRender and Microsoft PowerPoint].

The term biocompatibility is defined by D.F. Williams as “the ability of a material to perform with an appropriate host response in a specific application” [22]. The science community has discussed throughout the conception of biomaterials as to how to characterize biocompatibility of a material and to what degree. Through decades of research in both in vitro and in vivo models, the term can be subdivided into four individual categories which must be reviewed for a material to be labeled as biocompatible: (1) toxicology, (2) reaction to extrinsic microorganisms, (3) mechanical effect, and (4) cell–biomaterial interactions (Figure 2) [23]. This has led to the development of both in vitro and in vivo methods for the assessment of each subdivision, and the expansion of standardized procedures. These methods must answer with high certainty the public concern that a device or material will improve and ensure public safety, taking into consideration the type of biomaterial and biological components being assessed for cytotoxicity. This will determine any potential toxic mechanisms or material property effects that may occur due to reactions with interfering assay reagents, in order to prevent null or false readings. Most of these procedures are standardized by international agencies such as the International Organization for Standardization (ISO) and the American Society for Testing and Materials (ASTM), while other agencies will regulate or enforce these standards, such as the United States Food and Drug Administration (FDA) [24,25,26].

### 2.1. In Vitro Models

The evaluation of cytotoxicity in biomaterials can be simplified by observing how target cells or tissue will react in direct or indirect contact with biomaterials. If, under controlled conditions, cells react to the material with normal cell behavior and function (cell adhesion, proliferation, migration, and differentiation), we can conclude that the cell’s physiological system is normal or homeostatic. However, if under these same conditions, cells react with behaviors indicative of cell stress or apoptosis, we can conclude that the material induces some toxic or stressful effect on the cells. In vitro models have a distinct advantage of control over both the environment and the methods employed. Under stable laboratory conditions, specific variables related to cell–biomaterial cytotoxicity can be analyzed under conditions that provide more precise, accurate, and reproducible data to strengthen statistical models. Additionally, in vitro methods can result in both qualitative and quantitative data that assess the state of cellular health. Qualitative methods accomplish this by both evaluating the morphology of cell lines, after exposure to a material, and grading the health of cells based on the amount of cell death, cytoplasmic granule formations, and percentage of cells not adhered to the material. Quantitative methods determine the number of viable cells based on the presence of nucleic acids, metabolic by-products, or the expression of surface adhesion markers. These assessments follow the standards set by ISO, Section 10993, part 5, which stipulates the different methods for collecting cytotoxicity data *in vitro*, and can be further subdivided into more specific analyses of cell–biomaterial interactions [23,24,25,26,27].

The indirect contact method involves either Agar or Filter diffusion tests. In both cases, a barrier diffusion model is created when a monolayer cell culture is grown, and the physical biomaterial is placed in the same culture environment but separated by an Agarose gel or cell filtration film. Over time, the material may diffuse compounds through the membrane and into the culture where it will make direct contact with the cell line. The purpose of this experiment is to observe whether the cell lines can be induced towards a cytotoxic state by providing indirect exposure to potentially toxic materials, protein-loading components, nanomaterial agglomerates, and degradation agents. The advantage of this system is that it is controlled to examine the toxicity of cells specific to the leeched components from the materials. Meanwhile, qualitative and quantitative assays provide additional sensitivity and accuracy parameters for identifying the specific toxic components responsible. The disadvantage of this system is the omission of toxic effects due to mechanical trauma, mechanobiological pathways, and cell–biomaterial interactions. This must, instead, be further analyzed with a direct contact model [23,24,26,27].

The direct contact method involves the direct contact of cells and substrate, with no membrane separation. The cell line is cultured with the material, which adds the cytotoxicity variable of cell–biomaterial reactivity, as well as the physicochemical variables specific to indirect methods of analysis. There is additional variability in the nature of the materials (three dimensional (3D) structure, bioresorption, nanotopography, etc.) being evaluated and the different cell lines used, all of which cause concern in establishing standard testing methodologies for direct contact methods. The higher degree of variability creates a more complex model of analysis to determine what can elicit a toxic cellular response. Direct contact is a rapid, inexpensive, and sensitive method to assess these toxicity factors, but the variability makes it more difficult to determine with certainty what the exact causes of cytotoxicity are. Therefore, it is necessary to confirm the cause of cytotoxicity with additional testing. If the material under a direct contact model does not elicit a cytotoxic effect to the cell lines, then an initial conclusion would be that both the physicochemical and mechanical properties of the material are not cytotoxic to the cell lines. Although further confirmation with repeated direct contact tests utilizing different cytotoxicity assays is needed, the potential statistical power of a single direct contact test performed correctly could conclude with high certainty whether a material is cytotoxic or not [23,24,26,27].

The extract dilution (elution) test involves suspending a sample of the material in a physiological solution to extract elution solvents present in the material during the production phase. During this process, the solution will simultaneously minimize physical and chemical damage to the material and remain compatible with the cell lines. Elution samples are then added to monolayered cell cultures for 24–48 h before toxicity assays are introduced. Similar to the indirect contact method, the elution test often results in a consistent toxicity pattern in cell culture, due to minimal variability of elution solvents that are present in the material during production. Although this analysis is a good first approach to determine the cytocompatibility of the material, it suffers the same disadvantages as the indirect contact method [23,24,26,27].

Qualitative assessment of cytotoxicity in in vitro studies involves the use of categorical variables to determine if cytotoxicity is present in cell lines. This form of assessment will often use stains for viable and non-viable cells under fluorescent microscopy (FM) as a morphological indicator of cell health. Some of the more common viable stains involve high affinity towards specific cell structures, such as the nucleotides within the nucleus, adeno-triphosphate (ATP) and co-enzymes in the mitochondria, the permeability of the cell membrane, or cytoplasmic proteins/cytoskeletons. Fluorescent stains must be sensitive enough to differentiate between cell structures, or can be used in combination, such as the use of nuclear stains coupled with cytoplasmic counter stains. The intensity of the stain is correlated to the number of structures present in the cell, and the combination of stained cells gives an indication of viability for the entire culture. In addition to FM, viability data can be acquired using light microscopy (LM) or scanning electron microscopy (SEM) to determine cell morphology and death based on physical characteristics such as cell shape, size, spreading, or density. Cells suspended in media and incubated for 24–48 h, which exhibit spherical shapes and minimal surface spreading, are often key indicators of apoptotic behavior. While viability stains can confirm that cell integrity, deoxyribonucleic acid (DNA), and cytoplasmic environments are stable, morphological analysis is often categorized as a graded scale assessment of cytotoxicity. Other qualitative methods include real-time tracking of cells, which involves implanting fluorescent protein or radioactive isotope components, or labeling protein/gene sequence markers using fluorochrome-labeled antibodies. These specific markers can then be detected and quantified using spectrofluorometric analysis, scintillation to detect radio isotopes, or imaging software. Caution must be reserved when interpreting qualitative data, as they will often introduce debate among experts as to what the cells’ morphology indicates about the health of the cells. It is important to design in vitro models to account for statistical errors based on the sensitivity and accuracy of the assays used. This can be accomplished by including enough replicates to overcome these limitations and using experts to minimize the risk of bias observations. With a categorical data set, it is often difficult to determine whether a correlation exists between the material and variables for cytotoxicity, as compared with continuous data acquired during quantitative tests. This is why it is suggested that qualitative data be complemented by quantitative data to detect specific cytotoxic pathways and determine a correlation between the material variables and any cytotoxic mechanisms [26,28,29,30].

Quantitative assessment of cytotoxicity in in vitro studies involves methods that can assess the specific factors related to cell death. One of the most common quantitative biomaterial assessments is to count the number of viable cells across a material surface under normal incubation conditions. While the previously described colorimetric assays are common qualitative methods for assessing the proliferation pattern of cells, this method involves molecular compounds designed to fluoresce when exposed to cells with activated cytotoxic pathways. The fluorescent labeled cells can be quantified by counting the number of viable or non-viable cells through a hemocytometer or flow cytometer. The light intensity of the fluorochrome and density of the culture area can then be determined. The fluorescence is detected at a specific wavelength using calibrated light spectroscopy and imaging software (e.g., ImageJ) to determine the optical properties of images taken by FM. This is possible only if the fluorochromes remain bound to the internal cell structures and minimal leaching out of the cell is observed. One of the most reliable and sensitive calorimetry evaluations are tetrazolium-salt-based assays. The oxidation of nicotinamide adenine dinucleotide phosphate (NADPH) is measured to reduce tetrazolium salt into a fluorescent protein known as Formazan. This allows for the determination of mitochondrial metabolic activity with flavoproteins. Soluble Formazan, present in the surrounding media, can then be detected using a spectrometer apparatus where the fluorescence intensity can be correlated to give an estimated number of viable cells that is within an acceptable range. Each of these assays are labeled as 3-(4,5-dimethylthiazol-2-yl)-2,5-diphenyltetrazolium bromide (MTT), with other iterations often removing the extra step of solubilizing the formazan proteins into the media solution (e.g., 3-(4,5-dimethylthiazol-2-yl)-5-(3-carboxymethoxyphenyl)-2-(4-sulfophenyl)-2H-tetrazolium (MTS), water-soluble tetrazolium salt (WST), or 2,3-bis-(2-methoxy-4-nitro-5-sulfophenyl)-2H-tetrazolium-5-carboxanilide (XTT)). Calorimetry assays often consist of simple, efficient protocols, while also providing extremely sensitive and accurate data that correlate to the physical cell numbers. This presents possible limitations to their effectiveness around different cell lineages and any indirect methods of quantifying cell numbers. Some assays could be toxic to certain cell lines, such as those using radioactive isotopes (e.g., 3H-thymidine). Others may not take into consideration outside factors that trigger apoptotic pathways, leading to incorrect interpretation of the data. Biomaterials can also impact the function of these assays by either activating the fluorescence as a false positive or preventing the reagent from binding to its specific target as a false negative. It is therefore essential that the limitations of one assay be supported by the advantages of another assay. This will ensure that the data are interpreted correctly and provide a standard guide of compatible assays for different cell lines and biomaterials that can be utilized in vitro. Lastly, there are methods that involve detecting and quantifying more precise protein/genetic markers indicative of activated apoptotic pathways, such as pro-apoptotic genes (b-cell lymphoma 2 (bcl-2) associated x-protein (bax), bcl-2 antagonist/killer 1 (bak), and cytochrome c), and anti-apoptotic genes (bcl-2, bcl-extra-large (bcl-xl), and myeloid cell leukemia-1 (Mcl-1)). Western blot (WB), quantitative polymerase chain reaction (qPCR), and immunofluorescence (IF) are all methods capable of identifying apoptotic pathways initiated by a specific toxicity profile. The ratio of labeled surface markers, gene sequences, or proteins to the total image area or volume of cell culture can be quantified to indicate whether apoptotic behavior is present along one or more specific pathways [26,27,29,31,32,33].

### 2.2. In Vivo Models

While in vitro models are more simply designed to control experimental variable interactions and determine the correlations between them, they are incapable of recreating the same biological/physiological environment that the implant will be exposed to in a living organism. Therefore, the full spectrum of measurements of biomaterial performance cannot be assessed. In vivo models are a more complex system in which two or more experimental variables, including factors not used or relevant in the in vitro model, are now exposed. Inflammatory responses, degradation, biochemical kinetics, angiogenesis, and 3D tissue remodeling are complex processes that can only be fully studied *in vivo*. This can make opportunities to observe and identify associations between variables difficult. The goal of an in vivo model is to not only mimic the physiological conditions of a clinical case, but to obtain meaningful data that can be measured and analyzed for clinically relevant differences in material performance. It is prudent to observe overall physiological reactions and compare them to the current gold standard (positive control variable) and clinical care needed to determine the efficacy of the implant towards its intended targeted therapy. This essential step requires careful selection of the model system, variable control, and access, depending on the application of the implant and the current standardized ISO and ASTM guidelines for in vivo testing [34,35].

The use of mammalian physiological systems can accurately represent most of the conditions that biomaterials will be exposed to under practical clinical applications. Therefore, in vivo methods are the most reliable way of assessing how biomaterials will perform in a physiological environment that is similar to that of a human system. In an attempt to standardize in vivo models, we can tightly control variables such as anatomical location of implant site, size of the implant incision, species, gender, age, sample size, and methodologies for assessing material performance. The design of the model should narrow and strengthen cause/effect relationships of physiological events to the interaction of biomaterials. The controlled variables should also be focused towards answering specific questions related to the assessment of biomaterials in one or all domains of in vivo biomaterial assessments (biocompatibility testing, bioactivity testing, or preclinical testing). Meanwhile, these variables should retain the ability to translate these control parameters to alternative or more complex animal models [34,35].

*In vivo* assessment of biomaterials for neural regenerative applications must therefore fall under the same system of standardization for in vivo models. In an attempt to standardize, there have been numerous in vivo studies that evaluated peripheral nerve repair using scaffolds, creating a varied list of the most common animal model designs and methodologies for assessing nerve recovery. Literature reviews and studies of in vivo neural regeneration models found that the majority of these studies utilized rat models to assess scaffold performance, followed by mice and rabbits, respectively. Of these studies, the majority create defects in the sciatic nerve, with most defect gaps being between 5–10 mm. Additionally, the studies using rat models most commonly used quantitative histology analysis to assess nerve healing, followed by nerve morphometric analysis, electrophysiology, and immunohistology, respectively. The number of animal parameters to consider and the degree of control between model variables has created problems among investigators in establishing a consensus on the best animal model design and methods for assessing nerve recovery [34,35,36].

## 3. Polymers for Neural Applications

Numerous materials are available for biological applications in nervous tissues. Arguably, the most important characteristics are those that influence safety and efficacy. For a material to be used in the body, it must be deemed safe, biocompatible, and approved for use by the FDA. The FDA has an established set of guidelines that states that the biocompatibility of each material component should be understood at each step in its formation. This includes the original material before and after polymerization, as well as after sterilization, coatings, and the degradation products. Ultimately, the material should not result in an adverse biological response from the body [37]. A variety of materials have been established as biocompatible, such as the synthetic polymers polyglycolic acid (PGA) and polylactic acid (PLA), which are widely used in biodegradable sutures [38]. Others are more problematic, such as the synthetic polytetrafluorethylene (ePTFE), whose lack of degradability has resulted in long-term foreign body reactions [39]. All polymers have advantages and disadvantages which may differ among natural and synthetic polymers. Those most suitable for nerve tissue engineering will be discussed herein (Table 1).

### 3.1. Natural Polymers

Characteristics of natural polymers should include biocompatibility and biodegradability, minimal immunogenicity, and, ideally, provide properties that mimic the biological extracellular matrix (ECM) to provide structural and functional support. Replicating these properties allows for high-quality cellular interactions, including cell adhesion, proliferation, and differentiation for tissue regeneration. However, individual natural polymers tend to be less stable, have poor mechanical properties, and the purification method is less standardized. The variation between polymers and among different products of the same polymer is a potential limitation to translating natural polymers to commercial use because reproducibility is a critical factor in developing an optimal treatment. [15,39,40,41,42,43].

Collagen is an abundant natural polymer found in connective tissue and the ECM and is biocompatible, biodegradable, and bioactive. Currently, five FDA-approved collagen nerve conduits are commercially available. Many in vitro studies, in vivo studies, and clinical trials have identified the effective use of collagen support for wound healing, cell adhesion, migration, proliferation, and functional nerve regeneration. Additionally, collagen has been shown to bridge neural injury gaps up to 20 mm in length [13,39,44]. Collagen has tunable physical characteristics that can aid in directing axonal growth. However, collagen has limited mechanical stress resistance, which can be reinforced with additional biomaterials and/or fabricated into a hydrogel. Fabrication can be difficult due to collagen’s poor manipulability [41,42]. In some cases, collagen has decreased neuroma formation and scarring, but has not wholly eliminated these complications [19,42]. A recent study assessed mesenchymal stem cell (MSC) spheroid-loaded collagen hydrogels to promote neurogenesis and anti-inflammatory properties. Injuries treated with collagen hydrogels, with and without the MSCs, significantly improved cell viability, neural differentiation, and mediation of inflammatory components compared to negative controls [45].

Hyaluronic acid (HA) is a natural polymer found in the ECM of neuroepithelial-derived tissues. In addition to its biocompatibility, HA is known for reproducible outcomes and efficacy in decreasing scar tissue formation [19,42,46]. HA hydrogels are known to be cytocompatible with no significant immune response when co-cultured with human peripheral blood mononuclear cells in vitro [47]. However, its fast degradation and poor mechanical properties limit its solo use. Fortunately, HA can be combined with additional material to optimize its mechanical properties for neural applications [19,42,46].

Silk fibroin is a polypeptide isolated from the natural polymer silk. It is a strong, stable, biodegradable, and biocompatible material. It has also been shown to support cell attachment and neural and Schwann cell proliferation to promote myelinated axon regeneration. Silk fibroin has desirable elasticity and flexibility to avoid collapse, and effective permeability allowing for nutrient exchange. However, silk fibroin’s limitations result from its mechanically weak and fragile properties [42]. Silk fibroin can be processed using several different methods, including 3D bioprinting [39,40,41,48]. However, silk fibroin can be difficult to purify, as often is a natural polymer. A 2021 study by Lin et al. showed strong immune responses from their silk fibroin. After further experimentation, they discovered endotoxin contamination that adversely affected biocompatibility [47].

Fibrin is a natural polymer whose role in the body involves blood clotting. Fibrin has been shown to improve axonal regeneration and support blood vessel reparation [19]. Fibrin promotes cell adhesion for improved viability, proliferation, and differentiation of neural cells. Fibrin has been shown to prevent fibrous tissue formation and scarring while retaining biocompatibility [42]. Cell viability and neural differentiation have recently been reported after 3D printing (3DP) fibrin-based bioinks containing stem cells. Though rapid degradation limits its effectiveness, with adequate porosity for nutrient and waste exchange, and appropriate mechanical strength, fibrin-based bioink scaffolds achieved supportive results for neural applications [42,49,50].

Chitin is the second most abundant natural polymer on Earth [44]. However, due to its poor solubility, it is often modified to chitosan, maintaining the same chemical structure while adding necessary free amine groups [51]. Chitosan is low-cost, easy to produce, and biocompatible [52]. Although chitosan does not have desirable degradation and mechanical properties, chitosan has ECM-like features that support numerous studies showing significant improvements in functional axon regeneration, cell adhesion, and neural differentiation while decreasing scar formation in vivo [19,39,40,41,42,44,48,51]. A recent review on chitosan scaffolds for the treatment of myocardial infarctions has been reported, detailing polymer characteristics, such as elasticity and improved electrical conductivity, that would be translatable and beneficial for neural applications [53]. Meanwhile, a micropatterned, porous chitosan conduit was evaluated in a 10 mm sciatic nerve defect rat model. After three months, the chitosan conduit showed improved functional recovery comparable to or better than an autograft, though there was lesser neural, ECM, and blood vessel expression. The authors hypothesized that since chitosan can be easily modified, immobilizing growth factors through surface adsorption could improve this material facet [54]. One interesting disadvantage of this material is due to its sourcing. Since chitin is a core component of the exoskeleton of crustaceans, it may increase the risk of seafood allergy and inflammatory immune responses in certain patients [44,55].

There are additional natural polymers that, when combined with other materials, have been shown to promote axonal regeneration. Still, these polymers are less efficient when used alone due to weak mechanical resistance, fast degradation rate, and insufficient solubility. These materials include alginate, gelatin, and keratin, all of which, despite these challenges, have been shown to exhibit excellent biocompatibility [19,41,42,48].

### 3.2. Synthetic Polymers

Many synthetic polymers exhibit biocompatibility without cytotoxicity and can either be biodegradable (also known as bioresorbable), often preferred, or non-degradable. Poor degradation of synthetic polymers limits their use due to the higher potential for chronic inflammation [43]. These polymers also have uniquely beneficial characteristics, including tunability in mechanical characteristics and degradation rate. These polymers are easier to manufacture into complex architectures to better achieve biomimicry of the desired microenvironment, and are readily reproducible. One disadvantage is that these materials can be costlier and more time-consuming to produce. The most common synthetic polymers that support axon regeneration, guidance, and cell migration for improved nerve repair will be discussed [39,40,41,42,48,56].

Silicone and ePTFE are synthetic, non-degradable polymers that successfully treat PNIs. A second surgery to remove the implant may be required if complications, such as long-term foreign body reactions, occur. Despite this shortcoming, these polymers remain biocompatible for PNI healing and successful regeneration of myelinated axons [13,39,44].

Probably the most studied synthetic polymers for neural applications are polyesters. Polyesters include PGA, PLA, poly (lactic-co-glycolic acid) (PLGA), polycaprolactone (PCL), and poly(L-lactide-co-ε-caprolactone) (PLCL). These materials are favorable for use because of biocompatibility, tunable degradability, and limited cytotoxicity of degradation by-products. However, if the degradation occurs too rapidly, an acidic, inflammatory environment has been documented, resulting in tissue necrosis. Interestingly, PLGA’s degradation can be adjusted by changing the molar ratio of PGA to PLA, while PLCL’s copolymerization can neutralize the acidic environment [34,39,44,56,57]. Polyesters have desirable mechanical properties, flexibility, porosity, stability, solubility, hydrophilicity, crystallinity, and processability, making them valuable candidates for bioinks and 3DP methods. Yet, evidence of material deformation has been observed with long-term strain [20]. Many studies have shown their support for axon extension, regeneration, myelination, neural cell proliferation, differentiation, and neurotrophic factor expression [19,39,40,44,46,56,57,58,59]. Although the regenerative effects of these polyesters tend to be similar, PLGA has been reported to exhibit accelerated effects for more remarkable functional improvement over PCL and PLA [19].

Polyethylene glycol (PEG) is a biocompatible, synthetic polymer often used to adjust hydrogel swelling and mechanical properties [43,48,56]. PEG is often modified before use due to its easy processability, allowing for chemical modification and cross-linking, as well as its use in manufacturing methods such as 3DP [40]. PEG biodegrades poorly, limiting its use in vivo [48,60]. However, when modified, PEG can improve the microenvironment and support neural cell adhesion, proliferation, and differentiation [19].

Finally, polyurethane (PU) is a versatile synthetic polymer with adjustable mechanical and degradation properties. The Advincula group has reported PU/graphene nanocomposites to have potential for biomedical applications based on relatively stable and low toxicity when co-cultured with mammalian NIH-3T3 cells [61]. However, degradation by-products exhibit potential cytotoxicity [62]. Favorable characteristics of PU include fatigue resistance, flexibility, adhesion, durability, crystallinity, permeability, and anti-thrombotic properties. Together, these characteristics have been shown to promote myelination, anti-inflammatory responses, and functional axonal regeneration for nerve repair [19,40,43,44,56].

**Table 1:** Characteristics of natural and synthetic polymers for nerve tissue engineering.

Polymer	Advantages	Disadvantages	Ref.
***Natural Polymers***			
Collagen	Favorable biocompatibility, biodegradability, bioactivity, and processability.	Low mechanical stress resistance, often requiring additional materials or cross-linking. Poor manipulability can make this difficult. Evidence of neuroma scar formation.	[13,19,39,41,42,44,45]
Hyaluronic Acid (HA)	Favorable biocompatibility. High producibility. Preventative of scar formation.	The fast degradation rate and poor mechanical properties can be adjusted with additional materials.	[19,42,46,47]
Silk Fibroin	Strong, stable, biocompatible, and biodegradable. Favorable elasticity, flexibility, permeability, and processability.	It can be difficult to purify. Often mechanically weak and fragile.	[39,40,41,42,47,48]
Fibrin	Desirable biocompatibility and cell adhesion. Preventative of scar formation. Supports blood vessel reparation.	Rapid degradation rate.	[19,42,49,50]
Chitin, Chitosan	Desirable biocompatibility. Abundant, low cost, easy to produce. ECM-like behaviors.	Chitin has poor solubility, improved with Chitosan modification. Weak degradation and mechanical properties. May increase the risk of seafood allergy and inflammatory immune responses in certain patients.	[19,39,40,41,42,44,48,51,52,53,54]
***Synthetic Polymers***			
Non-degradable polymers (Silicone, ePTFE)	Biocompatible for PNI healing.	Lack of degradation requires a second removal surgery before long-term foreign body reaction.	[13,39,44]
Polyesters (PGA, PLA, PLGA, PCL, PLCL)	Favorable biocompatibility. Tunable degradability. Easily excreted by-products. Desirable mechanical properties, flexibility, porosity, stability, solubility, hydrophilicity, and crystallinity. Favorable processability.	If it degrades too rapidly, PGA and PLA cause an acidic, inflammatory environment, yet PLCL copolymerization can neutralize an acidic environment. Deformation with long-term strain.	[19,20,39,40,44,46,56,57,58,59]
PEG	Favorable biocompatibility. Easy processibility allows for chemical modification and cross-linking to tune hydrogels’ swelling and mechanical properties.	Poor degradative properties.	[19,40,43,48,56,60]
PU	Favorable biocompatibility. Tunable mechanical and degradation properties. Favorable fatigue resistance, flexibility, adhesion, durability, crystallinity, permeability, and anti-thrombotic properties.	Degradation by-products exhibit potential cytotoxicity.	[19,40,43,44,56,61,62]

## 4. Cells That Interact with Biomaterials

Regardless of the material, it is essential to consider the types of cells interacting with the surface when fabricating scaffolds. These cell types include any exogenous cells seeded on the construct and the endogenous cells within the injured area. Regenerative medicine for neural applications will often add primary culture Schwann cells, neural stem cells (NSCs), MSCs, embryonic stem cells (ESCs), or induced pluripotent stem cells (iPSCs) to scaffolds [19,43]. The source of these cells can be autologous, allogeneic, or xenogeneic [20]. Overall, the type of cell and how it will interact with the biomaterial surface influences its ability to repair neural injury. Meanwhile, the body will have systemic and localized reactions to the implanted material. Following the standard pattern of wound healing after a nerve is damaged, the foreign object implanted in the injured area will interact with peripheral immune cells, fibroblasts, progenitor cells, and the neural and glial cells that form the body’s nervous system [15,63,64]. The interactions between these cells and different biomaterials will determine if the entire construct is both biocompatible and capable of promoting functional nerve recovery.

Schwann cells have been studied in conduits developed to treat PNIs. They are effective at directing axon elongation, neurite outgrowth, and forming a new myelin sheath, but difficulties in sourcing and purification have limited their use [19,20]. Stem cells are more commonly studied, having exhibited promotion of repair in neural injuries when added to a biomaterial scaffold. iPSCs seeded onto a PLA/PCL co-polymer conduit accelerated peripheral nerve repair [65]. The iPSCs were pre-differentiated into Schwann cell lineage before implanting the conduit into a sciatic nerve defect mouse model. This process removed the issues with sourcing and purification while retaining the benefits for directing axon growth. They found that adding the iPSCs to their material significantly enhanced their mice’s functional recovery and promoted neural and myelin tissue histologically [65]. Similar to ESCs, because iPSCs are pluripotent, they have no impact of age or disease on their development. However, this benefit is weighed against the concern that these cells could become tumorigenic and immunogenic [20]. NSCs have been studied due to their potential to differentiate into neural and glial cell types, making them available for broader applications and removing the potential for developing into unwanted cell types that may worsen the injury [19]. Lastly, MSCs have the added characteristic of being easy to access. They can often be obtained from disposable or non-invasive tissues, such as adipose tissue and the umbilical cord, of both autologous and allogenic donors. MSCs also proliferate quickly and provide immunoregulatory factors to promote a microenvironment suitable for nerve repair [20]. Adipose-derived MSCs, 3D-printed into a fibrin-based bioink, expressed immature neural and dopaminergic neural markers *in vitro*, presenting potential therapeutic effects for NDs such as Parkinson’s Disease [50].

When a nerve injury occurs, or a scaffold is implanted, many cell types are recruited endogenously to repair the damage. Although inflammation is a natural response to injury, the harm comes when the inflammation is prolonged. Local proteins adsorb to the material surface, and this material–cell interface’s properties determine the immune response’s extent [66]. If the interactions at the interface are non-immunogenic, the body accepts the material, i.e., it is biocompatible, and the inflammatory response will not last longer than a few days. However, if the material is identified as a foreign body, the secretion of inflammatory cytokines and chemokines will initiate a response from the immune system [66,67,68]. Phagocytic cells, such as neutrophils, monocytes, local macrophages, and microglia, are attracted to these signals and migrate to the injured area [66,67,68,69,70]. These cells will remove cellular debris and pathogens while releasing reactive oxygen species (ROS) that cause additional damage [68]. However, the recruitment of these cells is necessary to help with regeneration, as they also secrete beneficial growth factors that promote tissue organization and angiogenesis [67,68,70]. The monocytes recruited to the area differentiate into macrophages. These macrophages attempt to engulf the scaffold but cannot phagocytize such a significant material and become stressed. The microenvironment becomes chronically inflamed, and the macrophages begin to morph together and form foreign body giant cells (FBGCs). A fibrous tissue encapsulates the material and attempts to expel the material from the body, rejecting the implant [66,69,70]. Although the innate immune cells are the first line of defense, the adaptive T cells and B cells often complement the system, recognizing specific antigens and aiding in the cascade of inflammatory factors [69,70,71]. When the inflammation begins to subside around the biocompatible scaffold, neural and glial progenitor cells will move through the area to regenerate and remodel the tissue. Depending on whether the injury occurs in the CNS or PNS, these cells will differentiate into neural and glial cells, including astrocytes, oligodendrocytes, or Schwann cells, to rebuild the nerve. Each cell type has a vital role in the nervous system that helps regulate neural activity and the exchange of nutrients in a healthy microenvironment. Finally, macrophages, fibroblasts, and endothelial cells will reconstruct the surrounding ECM and vasculature [66].

## 5. Biomaterial Modifications Influence Cell Behavior

Although these polymers have desirable biocompatibility, tunable degradation, and mechanical properties that benefit biological use, most polymers are not used without additional modifications. These polymers are altered to optimize their use in neural applications while retaining the benefits of their material. Nanomaterials, such as graphene or gold, are commonly incorporated due to their conductive capabilities [52,72,73,74,75,76,77]. Polymers also can be altered for a more specific application. For example, cross-linking polymers to create hydrogels are suitable for use in the CNS, specifically for TBIs, and hollow conduits and wraps are more suitable for use in PNIs and SCIs due to the shape of axons and the spinal cord. While there have been many studies on the effects of polymers, incorporating nanomaterials and cross-linking changes the material’s characterization and the behavior of the cells they interact with [56]. In all, the goal of these modifications remains the same, to develop a safe and functional treatment for neural injuries [15].

### 5.1. Hydrogel Formation

Hydrogels can be used for various applications, including nerve tissue engineering. They are often preferred for CNS injuries, particularly TBIs, where nerve guidance conduits would be too invasive. Defined as 3D cross-linked polymers with high water content and tunable properties, hydrogels can comprise individual polymers or composites of two or more materials. When forming a composite, the strengths and weaknesses of the unique polymers are combined [13]. Overall, hydrogels tend to induce minor inflammation, have favorable biocompatibility and biodegradability, but have weak mechanical properties [43,56,78,79]. The molecular weights and the concentrations of the distinct polymers, alongside the cross-linking method, can be used to alter the characteristics of the hydrogel. Standard cross-linking methods include ultraviolet (UV) light, pH, and temperature. Additionally, each of these factors can alter the physicochemical properties of the materials to produce a treatment optimized for the nervous system [19,42,47,79]. These properties include gelation time, swelling ratio, physical size, shape, structure, mechanical strength, stress, porosity, and surface charge and chemistry.

The gelation time is essentialbecause the hydrogel needs to have the appropriate thixotropic properties (injectability, shear-thinning, etc.) to be appropriately processed (3D printed, injected, etc.). A benefit of hydrogels for their use in CNS injuries is that they can fill irregularly shaped injury sites while providing the appropriate structural support required for nerve development [79]. However, without the appropriate gelation, a hydrogel can take hours or days to obtain the proper mechanical stability characteristic of this modification [80]. In a study using a photocurable, methacrylate, silk fibroin bioink, the raw silk fibroin bioink took several hours to gel, making it a less-than-ideal candidate for clinical use [78]. By adjusting factors such as treating with enzymes or salts, altering conditions including pH and temperature, and the cross-linking method, they decreased the hydrogels’ gelation time to minutes. The material will degrade once a hydrogel has solidified and reached its maximum swelling ratio due to water uptake [78,81].

In addition to the gelation kinetics, the physicochemical attributes of the hydrogel significantly influence cellular behavior at the tissue–material interface. Characteristics such as porosity, micro patterns, single vs. multi-channel, filament diameter, and surface roughness can all change cell adhesion, proliferation, axon guidance, and neural differentiation [42,82]. Hydrogels mimic the natural ECM, providing a microenvironment that allows for cell signaling and cell adhesion to promote nerve regrowth [83]. A porous hydrogel creates the space for increased cell–cell interactions and access to nutrients and supportive molecules [78]. Meanwhile, the surface microtopography can increase cell adhesion, by increasing the material surface area while providing guidance cues for axon and neurite growth to their target location [79,82]. One study compared micropatterned chitosan conduits to non-patterned, random conduits of the same material. Their results showed that the micropatterning significantly improved functional recovery as compared with the non-patterned conduit [54]. In another study, a low-stiffness hydrogel greatly improved nerve growth in a sciatic nerve injury rat model compared to more rigid hydrogels of the same material [84]. In general, a Young’s Modulus value of 0.1 to 1.0 kPa provides the appropriate stress/strain to promote neural differentiation favorably [79].

These characteristics of hydrogels, and the physical and chemical cues they provide, are proper modes for delivering cells, drugs, and other biological factors to aid nerve repair. The material encapsulates the factors of choice to support further the environment in which it is implanted [82,83]. One study immobilized umbilical cord-derived MSCs and neurotrophic factor fibroblast growth factor (FGF) onto a heparin–poloxamer hydrogel to repair SCI. The study found decreased cell apoptosis and increased mitochondrial function in vivo compared to hydrogels without the additional cells and factors [85]. Other factors can be added to reduce the aversiveness of the injured microenvironment and better support endogenous nerve repair. For example, a 2022 study by Jin et al. observed that urolithin-A-loaded hydrogels in PCL conduits exhibited anti-inflammatory, antioxidant, and immunomodulatory effects [86].

A large field of study is involved in incorporating conductive polymers into hydrogels to improve the electrophysiological function of the regenerating nerve. Nerves have natural electrical activity in their tissue environment, and introducing electrical components before functional nerve regeneration has been shown to improve cell adhesion, self-renewal, proliferation, differentiation, and signaling. The surface charge of the hydrogel, when made highly positive, can improve the electrostatic interactions with the negatively charged cell membrane, to further enhance cell growth and neural differentiation [87]. Electrical stimulation has also been shown to accelerate axon regeneration for faster healing. This occurs as neurons can continue transmitting information, forming new synapses to integrate themselves into functional neural circuits [42,75,88,89]. Poly(3,4-ethylenedioxythiophene) (PEDOT) is a common conductive polymer that is being incorporated for neuroregeneration. Other conductive biomaterials incorporated include polypyrrole, polythiophene, and polyaniline. These polymers are often hard to process and contain due to their brittleness, rigidity, and insolubility, though PEDOT has had the best success thus far [42,74,82,87,90]. A biocompatibility study of electrically stimulated PEDOT in the brain found that significant damage was created at higher current densities and could not be reversed. However, low pulse currents did not significantly diminish cell viability [91].

### 5.2. Incorporation of Nanomaterials

Nanomaterials are materials composed of particles that have nanoscale dimensions (<100 µm). These can include various materials, among which carbon-based nanomaterials, such as graphene and its derivatives and gold (Au) nanomaterials, are commonly used. These materials improve polymers’ strength, stiffness, electrical conductivity, topographical features, and surface area while retaining biocompatibility. However, they are not without their challenges. Nanomaterials can improve the loading efficiency, binding efficiency, and sustained release of biomolecules, such as growth factors for enhanced regeneration, or drugs for therapeutic drug delivery. These particles are often conductive and hydrophobic, so they can be challenging to handle as they aggregate together and poorly disperse [41,75,88,89,90].

Graphene has two common functionalizations that often are used for neuroregeneration: graphene oxide (GO) and reduced graphene oxide (rGO). GO has been shown to increase secretions of neurotrophic factors, neural differentiation, cell attachment and proliferation, and neurite outgrowth. rGO offers these same regenerative benefits alongside increased electrical conductivity. Graphene can be incorporated in several ways, including blending with a polymer, or coating a surface [19,48,57,74,90,92]. Previous studies evaluated the effect of GO-coated PLGA electrospun nanofibers immobilized with two neural growth factors. They found that adding GO decreased fiber diameter, altered the surface chemistry, enhanced the hydrophilicity, and improved the binding efficiency and sustained release of the two growth factors. These studies also found maintained or increased cell viability, proliferation, and neural differentiation in vitro and in vivo [93]. Graphene may have bacterial inhibiting properties, which not only aid in the influence of neural and glial cell behavior but also impact the injured area by preventing infection and long-term inflammation of the microenvironment [87].

Gold nanoparticles offer the same biocompatibility and enhanced neuroregenerative effects as graphene and its derivatives, while retaining the same issues with aggregation and dispersion [52,94]. The results of a chitosan conduit modified with Au nanoparticles were studied. The material maintained desirable biocompatibility while improving cell viability, adhesion, proliferation, and differentiation by increasing surface roughness, reducing inflammatory response, and influencing signaling factors and electrical conductivity [52].

## 6. Biomaterial Fabrication Methods

**Figure 3:**
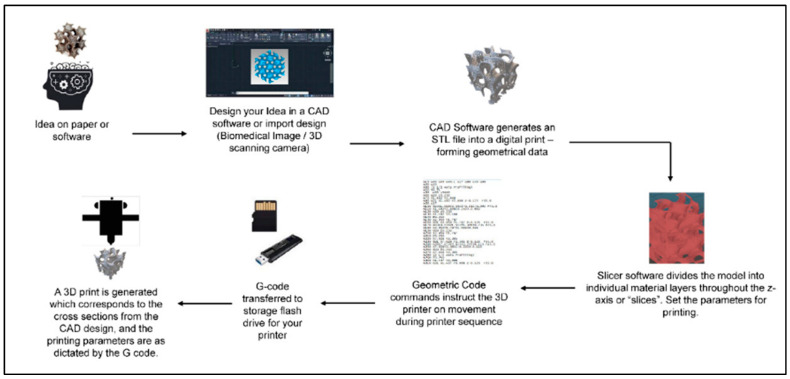
General schematic of the computer-aided 3DP process. Figure adapted from [95]. The use of this figure is governed by the Creative Commons Attribution (CC BY license https://creativecommons.org/licenses/by/4.0/) to the licensee MDPI, Basel, Switzerland.

Though there are many fabrication methods when it comes to materials for biomedical applications, 3DP has resulted in significant advancements in tissue engineering. One of the benefits of 3DP is its specificity. Computer-aided design (CAD), automated slicing software (creating a stereolithography (STL) file), and typed geometric code (G-code) allow for the creation of complex, biomimetic designs that match what is seen in tissues in the human body (Figure 3). 3DP creates these designs on demand, allowing for a future in personalized medicine [19,95]. Due to the complexities of the nervous system, 3DP is an excellent method to fabricate nerve guidance conduits and hydrogels for clinical use.

As discussed in previous sections, many properties contribute to biomaterials’ processability and printability. Biomaterials must be adapted for safety and efficacy in the body and withstand the fabrication, sterilization, and implantation processes. There is a similar process in the choice of the 3DP method. Although there are many types, specific techniques are more beneficial for bioprinting, and certain methods show favorable properties for printing live cells within bioinks. For example, the Advincula group has reported a number of studies using biomedically relevant polymer materials and composites for 3DP, mainly via the extrusion-based method of direct ink writing (DIW) [96,97,98,99,100]. The 3DP methods found to be best for nerve tissue engineering are photopolymerization techniques such as SLA and digital light projection (DLP), and extrusion-based techniques such as fused deposition modeling (FDM) and micro-extrusion [19,40,101]. Meanwhile, co-axial printing, an advanced extrusion-based technique, allows for a more complex, layered, multi-print head method that is favorable for live cell bioprinting [102].

SLA uses a UV laser to photosynthesize hydrogels or resins by immersing the stage in liquid and moving to change the layer height and resolution. Laser power, laser size, exposure time, and light wavelength can impact SLA printing. This method can be slow, costly, and dependent on specific material characteristics [19,43,101]. DLP is similar to SLA in that it uses photopolymerization. However, this method uses numerous mirrors to project the printed image, improving printing speed [43]. These photopolymerization methods are ideal for producing hydrogels for neural applications. Recently, a DLP 3D printer made photocurable silk fibroin hydrogels. Based on the data, any material with less than 10% wt./vol. hydrogel composition was difficult to print due to a loss in structural integrity. However, the hydrogel could be adjusted in a higher-range concentration (10–30%) to create physical properties, such as gelation time, swelling ratio, Young’s Modulus, porosity, and degradation time, that were optimal for nerve treatments. The authors also discussed maintaining approximately 95% cell viability when encapsulated inside the hydrogel. However, the fabrication process for making the materials, printing the structure, and implanting the material required 2 weeks, limiting application in clinical settings [78]. Another study, using a DLP 3D printer, printed a hydrogel loaded with a small flavonoid molecule, 7,8-dihydroxyflavone, to enhance the effects for a PNI treatment. The drug-loaded bioink was prepared within a day, and exhibited favorable mechanical properties and strong effects of cell adhesion and migration when co-cultured with Schwann cells [103].

FDM creates filaments using temperature changes to extrude material in a semi-liquid state, solidifying on a platform in a layer-by-layer deposition. This method is not ideal for direct cell printing if higher temperatures are required for the polymer(s). Although it is a simple, low-cost process, the prints may result in interlayer weakness depending on layer thickness, filament diameter, direction, and printing speed [19,43,49,101]. This is a standard method for printing nerve guidance conduits for PNI and SCI applications. In a study that used FDM to print PLA scaffolds coated with Au-nanoparticles, specific printing variables were kept standard, including layer height, fill density, fill pattern, extrusion temperature, bed temperature, and print speed. The study assessed variability in prints, including filament diameter, pore size, Young’s Modulus, and tensile strength. Slight variations were found, even though overall consistency between prints was considered favorable, while demonstrating biocompatibility and promotion towards neural lineage [94].

Micro-extrusion uses a continuous bioink stream dispensed through nozzles to fabricate structures using pistons, screws, or pneumatic pressures rapidly. Due to speed and force, this method can often exhibit too much shear stress for live cell printing, though adjustments made in these variables can exhibit favorable outcomes [19,49,101]. One study used a micro-extrusion bioprinter to print fibrin hydrogels containing MSCs. The MSCs showed high cell viability immediately after printing and 4, 9, and 12 days *in vitro*. The scaffold even promoted the successful differentiation of MSCs into neural and glial cell types and exhibited electrophysical/functional maturation in vitro [50].

These extrusion-based techniques have advanced to include co-axial printing, composed of multiple printheads with different parameters for different materials, that come together in alternating layers to create a single scaffold. One study cultured 3D ESCs differentiated towards spinal cord motor neurons, and suspended them within a GelMA/alginate hydrogel. First, PCL-based inner and outer hollow tubes were printed. The hydrogel was then printed between the two PCL walls, in alternating layers of ESC-loaded hydrogel, and alginate alone. Alternating materials and varying printing conditions between each material allowed for a construct that promoted cell viability and structural integrity, with properties promoting treatment of SCIs [104]. The alternating tissue layers were found to be more comparable to native tissues, and a beneficial method for bioprinting live cells [102]. Another study used two print heads, one with a fibrin-based bioink, and one with a cross-linker, so that the material was polymerized prior to extruding. This limits the shear stress of the live cells as they pass through the printing needle [50].

## 7. Future Perspectives

Future studies may look into additional methods to alter a material that could improve functionality for directing desired cell properties. Using existing materials, applications of more surface modification methods could be studied to aid in the control of protein adsorption, chemotaxis, and cell-tissue morphologies. These surface modifications include polymer grafting or self-assembly using chemical and physical adsorption methods [105]. Grafting polymers and polymer brushes could enable a more desirable surface functionality and chemistry, independent of the bulk thermo-mechanical properties of the individual biomaterials, to aid in cell proliferation, guidance of neural cell growth, and axon-dendrite distribution [106,107,108]. Further investigations of biomaterial surface morphology, thermomechanical properties (e.g., change in elasticity or flexural properties), directional properties, anisotropic properties, and processing methods that influence semi-crystallinity (e.g., polymer domain size) will bring us closer to improved processing and manufacturing methods for clinical applications.

The above methods show how a material can be modified to improve cell behavior to a specific biomaterial. Cell biologists and material scientists can work together to fabricate a surface according to the needs of the cellular applications. For instance, the importance of cell adherence is provided by a material’s surface topography and chemical composition. A surface functionalized with -COOH or -NH_3_ groups can enhance adsorption of proteins. Understanding and altering a material to influence neural lineage can drastically improve cell behavior for the appropriate applications. Conversely biological tools exist which can guide cells to behave as per their need [109,110,111,112].

The use of hyphenated and more state-of-the-art surface-sensitive spectroscopic and microscopic analytical characterization methods, such as advanced IR-AFM (infrared spectroscopy, atomic force microscopy) methods, higher resolution or super-resolution fluorescence microscopy, electrochemical-AFM imaging methods, and better TEM (transmission electron microscopy) and cryo-TEM methods, would advance the process of biomaterial characterization closer to physiological conditions for more accurate assessments [113,114,115]. At the same time, using probe methods and label or non-labeling methods (e.g., quantum dots) could enhance imaging to provide better and higher-throughput in interpretations [116]. The use of high-contrast imaging agents (dyes, nanoparticles, ligands, etc.) for multimodal and multiplexing is also important [117]. Finally, AI/ML (artificial intelligence, machine learning) methods can be applied to imaging modalities and polymer design methods. AI/ML could improve the testing methods and failure analysis of implanted materials [118].

**Figure 4:**
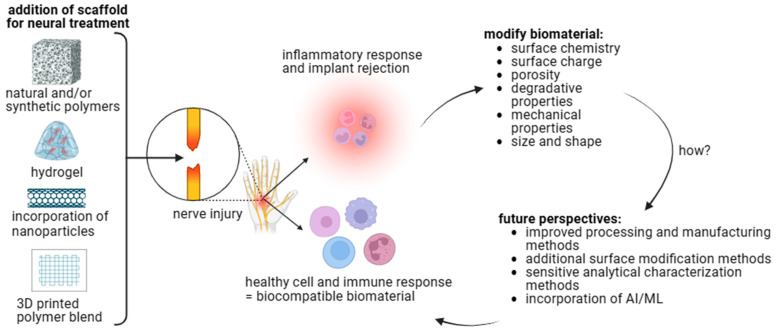
Biomaterial–cell interactions influence biocompatibility. Polymer choice, fabrication method, and additional modifications can alter the biomaterials’ physical and chemical properties. These properties affect cell behavior to determine local and systemic immune responses. Future perspectives should be considered to develop a therapeutic treatment for neural injuries [figure made with BioRender].

As summarized in Figure 4, recent progress in team science, technological advances in material science and cell biology, along with the novel opportunities at hand will provide potential tissue engineering products for neural repair.

## 8. Conclusions

Regenerative medicine and nerve tissue engineering are essential research fields for developing an effective treatment for neural injuries. Numerous natural and synthetic polymers exhibit excellent biocompatible, biodegradable, and bioactive properties, in addition to excellent processability and printability. The type of polymer, method of modification, and method of fabrication can significantly impact a polymer’s physicochemical properties. These properties, such as surface chemistry, mechanical strength, and topography, greatly influence cellular adhesion, migration, proliferation, and differentiation, impacting how the neural environment affects biocompatibility and regeneration. Although numerous factors are advantageous or disadvantageous, many of these polymers, modification techniques, and fabrication methods support the safe and effective regeneration of a physical and functional nerve. They are, ultimately, moving closer toward a treatment to improve the quality of life of the millions impacted by neural injuries.
